# Comparison of modified MAZE with minimally invasive monopolar ablation and traditional bipolar radiofrequency ablation in the treatment of atrial fibrillation

**DOI:** 10.1186/s13019-019-1012-x

**Published:** 2019-11-14

**Authors:** Wei Si, Sijia Yang, Linhui Pan, Chengchegn Li, Liang Ma

**Affiliations:** 1 Department of Cardiothoracic Surgery, First Affiliated Hospital of Zhejiang University, Hangzhou, China & School of Medicine, Zhejiang University, Hangzhou, China; 2 Department of Thoracic Surgery, First Affiliated Hospital of Zhejiang University, Hangzhou, China & School of Medicine, Zhejiang University, Hangzhou, China; 3 Department of General Surgery, First Affiliated Hospital of Zhejiang University, Hangzhou, China & School of Medicine, Zhejiang University, Hangzhou, China; 4 Department of Cardiothoracic Surgery, First Affiliated Hospital of Zhejiang University, Hangzhou, China & School of Medicine, Zhejiang University, Hangzhou, 310003 China

**Keywords:** Radiofrequency ablation, Atrial fibrillation, Bipolar, Monopolar

## Abstract

**Background and aim of the study:**

Atrial fibrillation (AF) is the most common atrial arrhythmia. Our aim was to compare the outcomes of atrial fibrillation treatment with original modified minimally invasive MAZE using monopolar radiofrequency ablation (mi-MAZE) and open surgery MAZE using bipolar radiofrequency ablation (os-MAZE).

**Methods:**

We searched the associated patients’ information on the medical record system of the First Affiliated Hospital, School of Medicine, Zhejiang University. Statistical Package for Social Sciences (SPSS) was used to analyse the data. The primary outcome is the atrial fibrillation ablation rate 3 months, 6 months, 12 months after operation. And secondary outcome is the postoperative quality of life.

**Results:**

The mi-MAZE group included 42 patients and the os-MAZE group had 65 patients. Three months after surgery, we found that 31 patients (77.5%) in the mi-MAZE group were sinus rhythm and 44 (71.0%) recovered sinus rhythm in the os-MAZE group. We followed up these patients on the phone or in person and scored them on the SF-36 scale. The results were found to be 120.2 ± 8.10 vs 110.6 ± 6.58 (mi-MAZE vs os-MAZE, *P* < 0.001).

**Conclusions:**

There was no significant difference of atrial fibrillation ablation rate (sinus rhythm recovery rate) between the mi-MAZE group and the os-MAZE group. The postoperative quality of life in mi-MAZE group was higher than that in os-MAZE group.

## Introduction

Atrial fibrillation (AF) is the most common atrial arrhythmia, with an activation frequency of 350–600 beats/min. The typical characters of AF are absence of clear P waves, presence of f waves, irregularly irregular ventricular rhythm and QRS complex < 0.12 s. Based on the Global Burden of Diease Database, the global morbidity rate of AF is 0.5% [1]. Thomas Wilke et al. showed a morbidity rate of atrial fibrillation of 2.132% through a study of 8.3 million patients in Germany [2]. The average age of these patients was 73.1 years old. A study in China showed that the overall morbidity rate of atrial fibrillation was 0.77% [3], and 0.65% after standardization according to the 2010 census results [4]. The risk of stroke and congestive heart failure in patients with atrial fibrillation is respectively 3 times and 5 times higher than that of the general population, and the patients‘quality of life is affected to some extent [5].

The etiology and pathophysiological mechanisms of atrial fibrillation are still unclear. The current mainstream hypothesis includes multiple wavelet re-entry hypothesis [6] and focal activation theory [7, 8]. The most common surgery of atrial fibrillation is the Cox-MAZE procedure, first reported by Cox et al. in 1989 [9–12]. The classic maze procedure is highly surgical technique dependent, with long operation time and high complication rates. Therefore a series of modified maze techniques, such as cryoablation [13], radiofrequency ablation, microwave ablation [14], etc., have been developed. The most currently used surgery is the modified type III MAZE.

Radiofrequency ablation (RF) mainly includes monopolar ablation and bipolar ablation. Martin-Suarez et al. compared bipolar radiofrequency ablation maze, monopolar radiofrequency ablation and interventional therapy [15]. They confirmed that the success rate of bipolar radiofrequency ablation maze is higher than that of monopolar and percutaneous intervention. Geidel et al. did a long-term follow-up of patients undergoing radiofrequency ablation, finding although the atrial fibrillation ablation rate in bipolar and monopolar RF modified maze was not significantly different, the operation time for bipolar radiofrequency ablation was significantly shorter than that of monopolar radiofrequency ablation, thus bipolar ablation is strongly recommended [16]. Besides the improvements in energy technology, the application of minimally invasive techniques has also improved MAZE procedure. The Wolf mini-MAZE is the most famous minimally invasive modified MAZE procedure. It is operated with a bipolar radiofrequency ablation forceps, assisted by thoracoscope through small incisions on both sides of the chest wall, with a continuous atrial fibrillation ablation rate of 85%. Although long-term follow-up results are unsatisfactory, the technique is less invasive and it does not need extracorporeal circulation [17]. Different from Wolf mini-MAZE, this study aimed to test the atrial fibrillation ablation rate and postoperative quality of life after the modified minimally invasive MAZE with monopolar radiofrequency ablation innovatively.

## Materials and methods

### Patients

The study included a total of 157 AF patients from two inpatient wards in the First Affiliated Hospital, School of Medicine, Zhejiang University. The minimally invasive MAZE group(mi-MAZE group) included 42 patients who underwent modified minimally invasive MAZE with monopolar radiofrequency ablation and mitral valve surgery from January 1, 2014 to May 31, 2018 (18 male: 42.9%, 24 female: 57.1%; age range: 27–70 years old), the open surgery MAZE group (os-MAZE group) includes 115 patients underwent traditional bipolar radiofrequency ablation and mitral valve surgery from January 1, 2014 to May 31, 2018 (78 male: 67.8%, 37 female: 32.2%; age range: 29–83 years old). Patients with a history of atrial fibrillation less than 2 years and a left atrial diameter < 60 mm with mitral valve disease were selected. Eventually 107 AF patients [42 patients in mi-MAZE group (100%), 65 patients in os-MAZE group (56.5%)] were included. All patients were diagnosed as AF by conventional 12-lead ECG (electrocardiogram) or 24-h Holter ECG. And echocardiography was used to diagnose mitral valve disease. The primary outcome is the atrial fibrillation ablation rate evaluated by ECG 3 months, 6 months, 12 months after operation. And secondary outcome is the postoperative quality of life, which is assessed by the SF-36 scale [18–20].

### Surgical technique

The mi-MAZE group: Our hospital used an original surgery technique for the mi-MAZE group—— After general anesthesia, incision was made in the fourth intercostal space of the right chest. After heparinization, peripheral extracorporeal circulation was established through the femoral artery and vein. Blunt dissection and monopolar ablation of the right superior and inferior pulmonary veins were done during extracorporeal circulation. The left upper and lower pulmonary veins are bluntly separated after heart arrest. The Medtronic Cardioblate flushing radiofrequency system which is connected to the monopolar ablation pen was used for ablation. Left atrial ablation path includes: left and right pulmonary vein ring, the line which connects right superior pulmonary vein and left superior pulmonary vein, the line which connects right lower pulmonary vein and left lower pulmonary vein, the line which connects the incision on interatrial groove and mitral annulus, the line which connects left lower pulmonary vein and mitral annulus, and the line which connects left atrial appendage and left superior pulmonary vein. After the ablation is completed, the valve is replaced or repaired and the left atrial appendage is ligated. The epicardial temporary pacing leads are placed routinely.

The os-MAZE group: After general anesthesia, traditional sternotomy was made in the midline. After heparinization, extracorporeal circulation was established through ascending aorta and right atrium. Blunt dissection and bipolar ablation of the right superior and inferior pulmonary veins were done during extracorporeal circulation and the Marshall ligament was cut off. The left upper and lower pulmonary veins are bluntly separated after heart arrest. The Medtronic Cardioblate flushing radiofrequency system which is connected to the bipolar and the monopolar ablation pen was used for ablation. Left atrial ablation lines were the same as in mi-MAZE group. The right atrium ablation lines include: the line which connects superior vena cava and inferior vena cava, the line which connects right atrial anterior wall incision, coronary sinus and tricuspid posterolateral annulus, the line which connects right atrial anterior wall incision and atrial septal fossa, the line which connects the tricuspid anterior leaflet and the right atrial appendage, and the line which connects the tricuspid posterior valve annulus and the incision on right atrial anterior wall. After the ablation is completed, the valve is replaced or repaired and the left atrial appendage is ligated. The epicardial temporary pacing leads were placed routinely.

The patients’ surgical approaches were determined by the surgeon.

### Follow up

All patients were treated with oral anticoagulation for 3 months. If the mitral valve replacement was not performed and the patient was in normal sinus rhythm, the oral anticoagulant was discontinued the fourth month after surgery.

Prevention of arrhythmia is one of the routine treatments. Amiodarone is used as the first choice: intravenous bolus injection of 300 mg, followed by continuous infusion of 1200 mg / 24 h until the first day after surgery. If the patients have no first degree AV (atrioventricular) block, oral administration of amiodarone of 200 mg is given three times a day until discharge. A maintenance regime of 200 mg / day is given for 3 to 6 months. In patients with contraindications to amiodarone, metoprolol or propranolol is administered. Diuretics and isosorbide mononitrate are also routinely used during hospitalization.

After their discharge, we followed up the patients by telephone, letter, and outpatient visiting. The effectiveness of the radiofrequency ablation was evaluated by the electrocardiogram and echocardiography 3 months, 6 months and 12 months after surgery. The postoperative quality of life was assessed by the SF-36 (short form 36 questionnaire) scale.

### Statistical analysis

Data were analyzed using SPSS (Statistical Package for Social Sciences, Microsoft) 22.0, using Shapiro-Wilk to test normally distribution. Differences between the mi-MAZE and the os-MAZE group were analyzed using Fisher’s exact test, Pearson’s test, continuous correction test for categorical variables and Student t test and Kolmogorov-Smirnov test for continuous variables. A *P* value < 0.05 was considered statistically significant for all analyses.

### Medical ethics

The study has been approved by the Ethics Committee of the First Affiliated Hospital, School of Medicine, Zhejiang University.

## Results

The preoperative baseline characteristics of the patients included in the study are shown in Table 1. Twenty-four women and 18 men were included in the mi-MAZE group, and the os-MAZE group included 46 women and 19 men (*P* = 0.148). The average age of mi-MAZE group was 56.8 ± 11.29 years and that of the os-MAZE group was 59.5 ± 10.04 years. Heart function was assessed using NYHA classification, and the overall difference between two groups was not significant. Of the 42 patients in the mi-MAZE group, 16 (38.1%) had hypertension, 25 (59.5%) had palpitations, 5 (11.9%) had type 2 diabetes mellitus, and 8 (19.0%) had pulmonary hypertension; One of the 65 patients in the os-MAZE group (1.5%) had COPD, 21 (32.3%) had hypertension, 28 (43.1%) had palpitations, and 14 (21.5%) had type 2 diabetes, 22 (33.8%) with pulmonary hypertension; there was no significant difference between the two groups. Preoperative cardiac ultrasonography measured LAD, LVDd, which were not significantly different. However, average LVEF was significantly lower and LVDs was significantly higher in the mi-MAZE group than that in the os-MAZE group (*P* = 0.001, Table 1). There was no significant difference of Euroscore between the two groups.

**Table 1:** The preoperative baseline characteristics of the patients

	mi-MAZE^f^(*n* = 42)	os-MAZE^g^(*n* = 65)	*P* value
Gender, F/M (%)	24/18(57.1/42.9)	46/19(70.8/29.2)	0.148^a^
Age, mean ± SD	56.8 ± 11.29	59.5 ± 10.04	0.201^b^
Functional capacity, *n* (%)			0.597^c^
NYHA class I	0(0)	0(0)	NA
NYHA class II	29(69.0)	38(58.5)	0.269^a^
NYHA class III	7(16.7)	15(23.1)	0.423^a^
NYHA class IV	6(14.3)	12(18.4)	0.573^a^
Type of atrial fibrillation			
persistent, *n* (%)	37(88.1)	58(89.2)	1.000^d^
paroxysmal, *n* (%)	5(11.9)	7(10.8)	1.000^d^
permanent, *n* (%)	0	0	NA
Chronic obstructive pulmonary disease, *n* (%)	0(0)	1(1.5)	1.000^e^
Hypertension, *n* (%)	16(38.1)	21(32.3)	0.539^a^
Palpitations, *n* (%)	25(59.5)	28(43.1)	0.097^a^
Type II diabete mellitus, *n* (%)	5(11.9)	14(21.5)	0.203^a^
pulmonary artery hypertension	8(19.0)	22(33.8)	0.096^a^
Preoperative cardiac ultrasonography, mean ± SD			
LAD(mm)	50.0 ± 6.09	48.1 ± 8.30	0.736^c^
LVDs(mm)	35.2 ± 5.42	31.9 ± 6.19	0.001^c^
LVDd(mm)	51.4 ± 6.90	49.1 ± 7.53	0.126^b^
LVEF(%)	59.3 ± 4.62	64.1 ± 8.13	0.001^b^
Surgical procedures, *n* (%)			
mitral vale replacement	29(69.0)	57(87.7)	0.018^a^
mitral valve plasty	13(31.0)	8(12.3)	0.018^a^
Euroscore, mean ± SD	4.33 ± 1.85	4.61 ± 2.05	0.867^c^

*Abbreviations*: *LAD* Left atrial diameter, *LVDd* Left ventricular diastolic diameter, *LVDs* Left ventricular systolic diameter, *LVEF* Left ventricular ejection fraction, *NYHA* New York Heart Association

^a^Pearson’s test

^b^Student T test

^c^Kolmogorov-Smirnov test

^d^Continuous correction test

^e^Fisher’s exact test

^f^ The modified minimally invasive MAZE with monopolar radiofrequency ablation group

^g^ The open surgery MAZE with traditional bipolar radiofrequency ablation group

During the operation, the mi-MAZE group had longer extracorporeal circulation time (Table 2), which may be related to thoracoscopic inconvenience. There was no significant difference in operative time between the two groups, indicating that the bipolar ablation group spent more time on the chest opening and closing than the monopolar ablation group. No adverse events occurred in both groups, such as myocardial infarction, acute renal insufficiency or death.

**Table 2:** Characteristics and events during the operation

	mi-MAZE(*n* = 42)	os-MAZE(*n* = 65)	*P* value
extracorporeal circulation, mean ± SD			
cardiopulmonary bypass (CPB) time(min)	114.5 ± 18.68	93.0 ± 21.18	0.000^a^
aortic cross-clamping (ACC) time(min)	77.0 ± 14.84	56.4 ± 16.63	0.000^b^
operation time, mean ± SD,(min)	211.1 ± 29.70	198.1 ± 31.74	0.104^b^
Intraoperative adverse events, *n* (%)			
Low cardiac output	0(0)	0(0)	NA
Myocardial infarction	0(0)	0(0)	NA
Acute renal insufficiency	0(0)	0(0)	NA
Death	0(0)	0(0)	NA
Conversion to open surgery	0(0)	0(0)	NA

^a^Student T test

^b^Kolmogorov-Smirnov test

42 (100%) patients in the mi-MAZE group and 65 (100%) patients in the os-MAZE group recovered sinus rhythm when the operation finished. The two groups also had similar ICU stays and hospital stays (Table 3). During hospitalization, 7 of 42 patients in the mi-MAZE group (16.7%) had recurrent atrial fibrillation, 4 (9.5%) developed atrioventricular block, 8 (19.0%) developed pulmonary infection, and 3 (7.1%) had a second operation including one with thoracotomy for hemotasis and another for a pacemaker implantation. Nineteen of the 65 patients in the os-MAZE group (29.2%) had recurrent AF and 6 (9.2%) had pulmonary infection. Methionine, digoxin, L-carnitine, metoprolol, isosorbide mononitrate, diuretics and other drugs were routinely used during hospitalization (Table 3). All patients were discharged uneventfully.

**Table 3:** Adverse events and medicine between the two groups during hospitalization

	mi-MAZE(n = 42)	os-MAZE(n = 65)	*P* value
Postoperative recovery of sinus rhythm, *n* (%)	42(100)	65(100)	NA
ICU stays, mean ± SD, (day)	3.1 ± 1.22	3.7 ± 1.37	0.721^a^
Hospital stays, mean ± SD, (day)	15.6 ± 4.37	16.7 ± 4.72	0.223^e^
Incident during hospitalization, *n* (%)			
Recurrent atrial fibrillation	7(16.7)	19(29.2)	0.139^d^
Atrioventricular block	4(9.5)	2(3.1)	0.325^b^
Second operation	3(7.1)	3(4.6)	0.901^b^
Stroke	0(0)	0(0)	NA
pulmonary infection	8(19.0)	6(9.2)	0.141^d^
CRRT	0(0)	0(0)	NA
Medication during hospitalization, *n* (%)			
Amiodarone	40(95.2)	59(90.8)	0.630^b^
Digoxin	31(73.8)	49(75.3)	0.855^d^
Levocarnitine	30(71.4)	51(78.5)	0.408^d^
Metoprolol	7(16.7)	10(15.4)	0.859^d^
Isosorbide mononitrate	37(88.1)	53(81.5)	0.365^d^
Diuretic	42(100)	65(100)	NA
Normal discharge	42(100)	65(100)	NA

*Abbreviations*: *CRRT* Continuous renal replacement therapy, *ICU* Intensive care unit

^a^Kolmogorov-Smirnov test

^b^Continuous correction test

^c^Fisher’s exact test

^d^Pearson’s test

^e^Student T tese

**Fig. 1:**
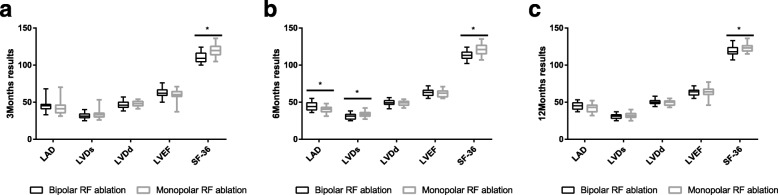
Echocardiographic and ECG data between the bipolar RF ablation group and the monopolar RF ablation 3 months, 6 months and 12 months after operation (the units of LAD, LVDs, LVDd are (mm); the units of LVEF are (%); the units of SF-36 are (score)). *P < 0.05. LAD, left atrial diameter; LVDd, left ventricular diastolic diameter; LVDs, left ventricular systolic diameter; LVEF, left ventricular ejection fraction; SF-36, short form 36 questionnaire

After surgery, we followed up 40 patients (2 patients had wrong telephone numbers) in the mi-MAZE group and 62 patients (3 patients withdraw) in the os-MAZE group. It was found that 31 patients (77.5%) in the mi-MAZE group were sinus rhythm 3 months after operation. However, 1 of the 31 patients became atrial flutter 6 months after operation. After treatment in hospital, he eventually recovered (Table 4). In the os-MAZE group, 44 (71.0%) recovered sinus rhythm 3 months after operation. After that, 2 more patients became atrial flutter (Table 4). Echocardiographic data showed no significant differences in LAD, LVDs, LVDd, and LVEF between the two groups 12 months after operation (Table 4 and Fig. 1c). We followed up these patients on the phone or in person and scored them on the SF-36 scale. The results 3 months after operation were found to be 120.2 ± 8.10 and 110.6 ± 6.58 (*P* < 0.001, Table 4). And the 12 months results were 123.5 ± 6.09 vs 119.3 ± 6.25 (mi-MAZE group vs os-MAZE group, *P* = 0.028, Table 4). The higher the score, the better the quality of life after surgery.

**Table 4:** Echocardiographic and ECG data between the two groups 3, 6, and 12 months after operation

	mi-MAZE(*n* = 40)	os-MAZE(*n* = 62)	*P* value
3 months after operation			
sinus rhythm, *n* (%)	31(77.5)	44(71.0)	0.465^a^
cardiac ultrasonography, mean ± SD			
LAD(mm)	41.5 ± 7.39	44.4 ± 5.84	0.069^b^
LVDs(mm)	33.4 ± 5.14	31.7 ± 3.78	0.170^b^
LVDd(mm)	48.0 ± 3.49	46.2 ± 4.43	0.055^c^
LVEF(%)	60.1 ± 6.16	62.7 ± 5.52	0.293^b^
SF-36, mean ± SD	120.2 ± 8.10	110.6 ± 6.58	0.000^b^
6 months after operation			
sinus rhythm, *n* (%)	30(75.0)	42(67.7)	0.266^a^
cardiac ultrasonography, mean ± SD			
LAD(mm)	40.1 ± 3.89	44.4 ± 4.95	0.001^b^
LVDs(mm)	33.8 ± 3.46	30.8 ± 3.72	0.010^b^
LVDd(mm)	48.6 ± 3.46	49.4 ± 3.56	0.318^c^
LVEF(%)	61.6 ± 4.03	62.8 ± 4.22	0.182^c^
SF-36, mean ± SD	121.4 ± 7.58	113.2 ± 5.36	0.000^b^
12 months after operation			
sinus rhythm, *n* (%)	31(77.5)	42(67.7)	0.164^a^
cardiac ultrasonography, mean ± SD			
LAD(mm)	41.9 ± 5.35	44.8 ± 4.67	0.070^b^
LVDs(mm)	32.1 ± 3.57	30.6 ± 2.98	0.182^b^
LVDd(mm)	49.1 ± 3.38	50.1 ± 3.03	0.129^c^
LVEF(%)	64.2 ± 5.53	63.3 ± 4.29	0.347^c^
SF-36, mean ± SD	123.5 ± 6.09	119.3 ± 6.25	0.028^b^

*Abbreviations LAD* Left atrial diameter, *LVDd* Left ventricular diastolic diameter, *LVDs* Left ventricular systolic diameter, *LVEF* Left ventricular ejection fraction, *SF-36* Short form 36 questionnaire

^a^Pearson’s test

^b^Kolmogorov-Smirnov test

^c^Student T test

**Fig. 2:**
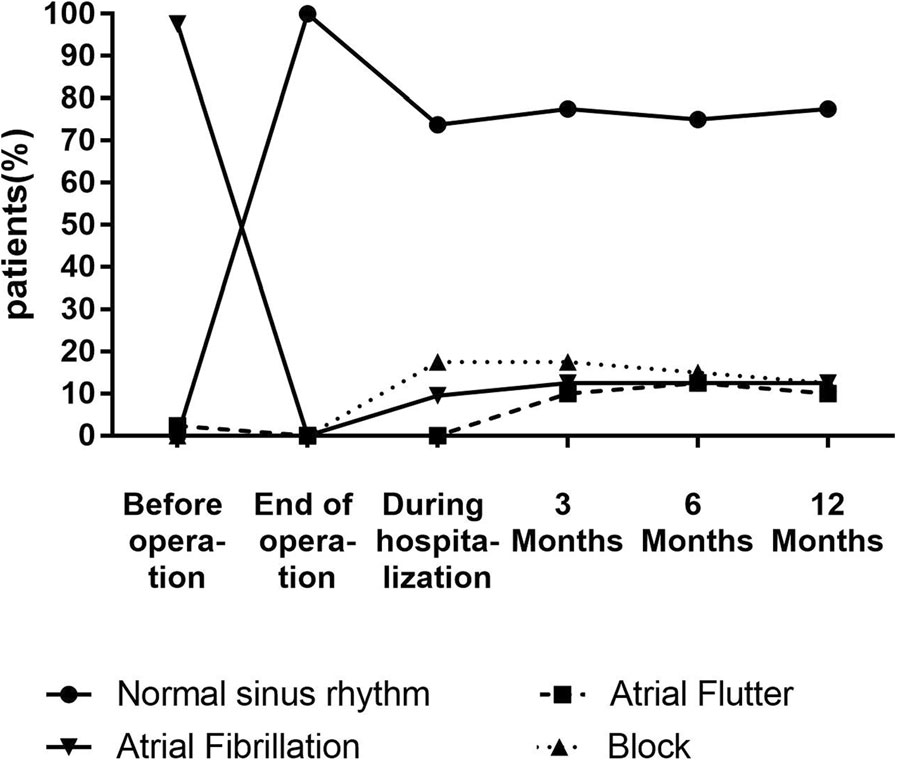
The percentages of normal sinus rhythm in the mi-MAZE group during hospitalization and follow-up

The rate of normal sinus rhythm in the mi-MAZE group before operation was significantly different from that of the end of operation (Fig. 2). And the rates were similar 3 months, 6 months and 12 months after operation (Fig. 2).

## Discussion

The MAZE procedure was first reported by Cox et al. in 1989 [9–12]. The basic technique was to use a cut-suture technique to separate the myocardium with multiple lines to make it to accept the activation of sinus node, while the atrial fibrillation wave could not be expanded. All of type I-III maze procedures use traditional cut-suture techniques to ablate atrial fibrillation. The type III maze is improved on the basis of type I maze, which reduces the risk of dysfunctional insufficiency and node conduction caused by type I and type II maze. To date, type III maze still has the highest atrial fibrillation ablation rate and is therefore the gold standard for atrial fibrillation ablation.

This study reached the following conclusions by comparing the baseline characteristics of the mi-MAZE group and the os-MAZE group before, during, and after surgery, and the results of 3 months’ follow-up. First, there was no significant difference in the rates of sinus rhythm between the mi-MAZE group and the os-MAZE group (77.5% vs 71.0%, *P* = 0.465, Table 4) 3 months after surgery, which means there was no significant difference in the surgical outcome between the two procedures. This result is consistent with the findings of the study by Geidel et al. [16] As we all know, the Wolf mini-MAZE is the most famous minimally invasive modified MAZE [17]. It is operated with a thoracoscope-assisted bipolar radiofrequency ablation clamp through small incisions on both sides of the chest wall. The postoperative atrial fibrillation ablation rate is 85%. Although long-term follow-up results are less than ideal, the technique has the advantage of less invasive and no need for extracorporeal circulation. Our minimally invasive monopolar radiofrequency ablation modified MAZE is different from the Wolf mini-MAZE. The surgical procedure used a monopolar ablation pen assisted by thoracoscopy, with extracorporeal circulation still required (because of mitral valve surgery). Compared with the study by Wolf et al., our minimally invasive monopolar radiofrequency ablation modified MAZE procedure resulted in a postoperative sustained atrial fibrillation ablation rate of 77.5% 12 months after operation.

In addition, this study showed that echocardiographic data was not significantly different between the two groups 12 months after the surgery. In addition, we used the SF-36 scale to assess the quality of life of patients and found that the quality of life score was significantly higher in the mi-MAZE group 12 months after operation (*P* = 0.028, Table 4). This indicates that the mi-MAZE group has greater quality of life, which includes the incision is easy to heal and the recovery is better. Although the extracorporeal circulation time of the monopolar ablation group was significantly higher than that of the bipolar ablation group, there was no significant difference in the overall operation time between the two groups, nor in the proportion of adverse events during hospitalization. Therefore, we recommend a minimally invasive monopolar radiofrequency ablation modified MAZE operation for the patients who had a history of atrial fibrillation less than 2 years with a left atrial diameter < 60 mm combined with mitral valve diseases.

There are still some limitations in our research, such as the small sample size, which makes the research results less confirmative. The mi-MAZE procedure has been started since 2014 in the Department of Cardiothoracic Surgery, First Affiliated Hospital of Zhejiang University. We have already collected as many patients as we could. Secondly, limited to one center, regional differences cannot be considered. Thirdly, since the latest mi-MAZE was in May 31, 2018, the longest follow-up period for this patient is only 12 months, which is still long enough for us to focus on primary and intermediate effectiveness of the two surgeries. Baseline balance was achieved as far as possible in patients before receiving surgeries, but there were still some significant differences. For example, preoperative LVEF was higher in the mi-MAZE group. Later, prospective studies should be carried on to explore the effects of minimally invasive monopolar radiofrequency ablation modified MAZE surgery.

This study is intended to evaluate the effectiveness of monopolar radiofrequency ablation in atrial fibrillation and the safety and superiority of minially invasive procedure. We mainly focus on primary and intermediate effectiveness of mi-MAZE and os-MAZE, and also on the differences of postoperative complications and quality of life between minimally invasive procedure and traditional sternotomy.

## Conclusion

There was no significant difference in atrial fibrillation ablation rates (sinus rhythm recovery rates) between the minimally invasive MAZE with monopolar radiofrequency ablation group and the open surgery MAZE with bipolar radiofrequency ablation group. The atrial fibrillation ablation rate of the mi-MAZE group achieved 77.5%. For the 3, 6, and 12 month postoperative SF-36 score, the mi-MAZE group was always significantly higher than the os-MAZE group. If a patient had a history of atrial fibrillation less than 2 years and with a left atrial diameter < 60 mm combined with mitral valve diseases, the minimally invasive monopolar radiofrequency ablation modified MAZE operation would be recommended.

## Data Availability

Please contact author for data requests.

## Abbreviations

ACC: Aortic cross-clamping
AF: Atrial fibrillation
AV: Atrioventricular
CPB: Cardiopulmonary bypass
CRRT: Continuous renal replacement therapy
ECG: Electrocardiogram
ICU: Intensive care unit
LAD: left atrial diameter
LVDd: Left ventricular diastolic diameter
LVDs: Left ventricular systolic diameter
LVEF: Left ventricular ejection fraction
mi-MAZE: Modified minimally invasive MAZE using monopolar radiofrequency ablation
NYHA: New York Heart Association
os-MAZE: Open surgery MAZE using bipolar radiofrequency ablation
RF: Radiofrequency ablation
SD: Standard deviation
SF-36: Short form 36 questionnaire
SPSS: Statistical product and servise solutions
